# Cytokine response in patients with chronic infections caused by *Staphylococcus aureus* strains and diversification of their Agr system classes

**DOI:** 10.1007/s10096-012-1633-7

**Published:** 2012-05-26

**Authors:** A. Szkaradkiewicz, T. M. Karpiński, A. Zeidler, A. K. Szkaradkiewicz, H. Masiuk, S. Giedrys-Kalemba

**Affiliations:** 1 Department of Medical Microbiology, University of Medical Sciences in Poznań, Wieniawskiego 3, Str., 61-712 Poznań, Poland; 2 Department of Conservative Dentistry and Periodontology, University of Medical Sciences in Poznań, Poznań, Poland; 3 Department of Microbiology and Immunology, Pomeranian Medical University, Szczecin, Poland

## Abstract

This study aimed to describe the levels of circulating cytokine levels produced by Th lymphocytes (IFN-γ, IL-4, IL-10, IL-17A), as well as the levels of cytokines produced by monocytes/macrophages (TNF-α, IL-1β, IL-12), in patients with chronic infections caused by *Staphylococcus aureus* strains, particularly in the context of the diversification of their Agr system classes. The studies were conducted on adult patients, including 50 patients with chronic suppurative dermatitis, 40 patients with chronic infections of the upper respiratory tract and 25 healthy individuals (control group). Blood serum cytokine levels were measured by enzyme-linked immunosorbent assay (ELISA). *S. aureus* was detected in cultures of suppurative dermal exudates or of pharyngeal smears. Classes of Agr systems in the *S. aureus* strains were identified using polymerase chain reaction (PCR). In both groups of patients, on average, levels of IFN-γ were doubled, while levels of IL-17A were increased by 2.5-fold, which, however, was not accompanied by increased levels of TNF-α or IL-12. The data indicate that the development of *S. aureus* infection among the studied patients was linked to an impoverished cytokine response of monocytes/macrophages, while that induced by the pathogen lymphocytes Th17/Th1 may be responsible for promotion of the chronic inflammatory response. In parallel, no quantitative or qualitative differences were disclosed between cytokine responses manifested by subgroups of patients infected with *S. aureus* strains belonging to class IV Agr, as compared to patients infected with strains of classes I to III Agr. Nevertheless, in the patients, strains belonging to class IV Agr prevailed, which points to the preferential relationship between the class and the pathogenicity of *S. aureus*.

## Introduction

The propensity of humans to develop community-acquired chronic infections with *Staphylococcus aureus* is well known and is most frequently manifested by chronic suppurative dermatitis or chronic infection in the upper respiratory tract. Therapy of such conditions with antibiotics used to be ineffective and linked to a persisting infection with *S. aureus* [1–4]. The role of immune reactions in the pathogenesis of chronic *S. aureus* infections still remains unclear. In the immune reaction, a very significant role is played by the cytokine response of lymphocytes Th and of monocytes/macrophages [5]. In turn, the pathogenicity of *S. aureus* is determined by the production of several virulence factors, in the pathogen remaining under the control of the global regulatory system Agr (accessory gene regulator), encoded by the *agr* locus [6–9]. Polymorphism of the *agr* locus allows to distinguish four major different classes of Agr system, referred to as Agr-I, Agr-II, Agr-III and Agr-IV. In parallel, their relationship is examined in relation to the type of human disease caused by *S. aureus* [10–12]. Considering the above, this study aimed to evaluate the circulating cytokine levels, mainly those produced by subpopulations of lymphocytes T helper (Th): interferon (IFN)-γ, interleukin (IL)-4, IL-10 and IL-17A, as well as of cytokines, mainly those produced by monocytes/macrophages: tumour necrosis factor (TNF)-α, IL-1β and IL-12, in patients with chronic staphylococcal infection in the context of diversification between Agr classes among the clinically isolated strains of *S. aureus*.

## Materials and methods

### Patients

The studies were performed in the Department of Medical Microbiology, University of Medical Sciences in Poznań and in the Department of Microbiology and Immunology, Pomeranian Medical University in Szczecin, Poland, over a period of two years (2010–2011). All the research protocols were reviewed and approved by the Ethics Committee at the University of Medical Sciences in Poznań, Poland.

A total of 90 adult patients with chronic suppurative dermatitis or chronic infection in the upper respiratory tract induced by *S. aureus* (referred by dermatologists and otolaryngologists) were recruited into the study. Additionally, 25 healthy individuals without bacteriological evidence of *S. aureus* infection served as a control group. Taking into account clinical data and the results of bacteriological tests, three study groups were distinguished. Group 1 included 50 patients (28 men, 22 women, age range: 18–52, mean: 35.3 ± 9.2 years) with exacerbation or a relapse of chronic suppurative dermatitis, manifested at least within the previous 6 months before inclusion into the study by chronic folliculitis or a relapsing furunculosis with isolation from the suppurative dermal exudate of *S. aureus*. Group 2 included 40 patients (18 men, 22 women, age range: 18–52, mean: 36.4 ± 9.4 years) with exacerbation of chronic infection in the upper respiratory tract, manifested at least within the previous 6 months before inclusion to the studies of chronic rhinopharyngitis or chronic pharyngitis, in the throat smears of whom the presence of *S. aureus* was disclosed. Group 3 (control) included 25 patients (11 men, 14 women, age range: 18–50, mean: 33.6 ± 9.4 years) in whom bacteriological examination of nasal and pharyngeal smears failed to demonstrate the presence of *S. aureus*. Moreover, dental examinations of patients failed to find any potential infection foci in the oral cavity. Within the previous 2 weeks, none of the patients in the mentioned groups were administered with antibiotics/chemotherapeutic agents locally or systemically. The material for studies involved nasal and pharyngeal smears, exudate from suppurative skin lesions and samples of peripheral blood.

### *S. aureus* detection

The bacteria were isolated on sheep blood agar within 20–24 h at a temperature of 37 °C in aerobic conditions. The developed colonies were subsequently identified as coagulase-positive staphylococci using conventional techniques (colony morphology, evaluation of haemolysis, staining according to Gram, production of coagulase, catalase, ability to decompose mannitol in Chapman medium). Final identification of *S. aureus* was conducted using the automated system ATB with the application of ID 32 Staph strips (bioMérieux). In the examined materials, the presence of no other pathogens was disclosed.

### Purification of DNA

DNA was isolated from the obtained isolates of *S. aureus* clinical strains. At first, the samples were digested with lysostaphin (10 μl of 1 mg/ml solution) and incubating them for 10 min at a temperature of 37 °C. Subsequently, 20 μl of proteinase K was added to each sample and, following mixing, the samples were incubated for 20 min at a temperature of 37 °C.

For the isolation of DNA, Sherlock AX (A&A Biotechnology, Gdynia, Poland) kits were used. The isolation of DNA was conducted as recommended by the manufacturer. The purified DNA was stored at −20 °C until further analyses were performed.

### Detection of Agr classes

The Agr classes were determined by two duplexes of polymerase chain reaction (PCR) according to the method of Shopsin et al. [11]. Since the PCR product sizes of Agr classes I and III and classes II and IV were similar, two duplex PCR reactions were performed. The reverse primers I (product size: 440 bp) and IV (product size: 588 bp) were used in the first reaction, and primers II (product size: 572 bp) and III (product size: 406 bp) were used in the second reaction.

PCR was performed in the Mastercycler gradient thermal cycler (Eppendorf). Aliquots of amplified samples (10 μL) were analysed by electrophoresis on a 1 % agarose gel and stained with ethidium bromide.

### Determination of cytokines

Cytokines were estimated in patient sera using an immunoenzymatic technique (enzyme-linked immunosorbent assay, ELISA). The tests took advantage of the following kits: for IFN-γ: Human IFN-γ High Sensitivity ELISA kit (eBioscience), manifesting a minimum detection dose (MDD) of 0.06 pg/ml; for IL-4: Quantikine HS Human IL-4 Immunoassay kit (R&D Systems), manifesting an MDD of 0.11 pg/ml; for IL-10: Quantikine HS Human IL-10 Immunoassay kit (R&D Systems), manifesting an MDD of 0.5 pg/ml; for IL-17A: Platinum Human IL-17 ELISA kit (eBioscience), manifesting an MDD of 0.5 pg/ml; for TNF-α: Quantikine HS Human TNF-α/TNFSF1A Immunoassay kit (R&D Systems), manifesting an MDD of 0.106 pg/ml; for IL-1β: Quantikine HS Human IL-1β/IL-1F2 Immunoassay kit (R&D Systems), manifesting an MDD of 0.057 pg/ml; for IL-12: Human IL-12p70 High Sensitivity ELISA kit (eBioscience), manifesting an MDD of 0.1 pg/ml.

The tests were performed as recommended by the manufacturers. Values of absorbance, depending on the estimated cytokine, were read at a wavelength of A = 450 nm or 490 nm using Reader 250 (bioMérieux). The results were obtained from standard curves.

### Data analysis

In the comparative analysis of cytokine levels in the studied groups, the non-parametric test of Kruskal–Wallis with Dunn’s test was employed.

In the analysis of Agr class systems in the examined strains of *S. aureus*, Student’s *t*-test for fractions was employed, indicating a difference between two structural indices.

In every test, hypotheses were verified at the significance level of *p* = 0.05.

## Results

Levels of IFN-γ, IL-4, IL-10 and IL-17A cytokines, produced by lymphocytes Th, detected in the sera of patient groups with *S. aureus* infections, manifesting by chronic suppurative dermatitis (group 1), chronic inflammation of the upper respiratory tract (group 2) and in healthy individuals are listed in Table 1. In group 1, the mean levels of IFN-γ and IL-17A amounted to 0.98 ± 0.55 pg/ml and 4.93 ± 1.54 pg/ml, respectively. In group 2, the mean levels of IFN-γ and IL-17A amounted to 0.87 ± 0.51 pg/ml and 4.87 ± 1.57 pg/ml, respectively. The levels manifested no significant differences between patient groups 1 and 2, but they were significantly higher than those in group 3 (control). In turn, levels of IL-4 and IL-10 did not differ from those in the control group.

**Table 1:** Levels of cytokines produced by lymphocytes Th (IFN-γ, IL-4, IL-10, IL-17A) in the sera of patients with chronic suppurative dermatitis (group 1), with chronic inflammation in the upper respiratory tract (group 2) and in the control group (group 3)

Studied cytokine	Mean values in pg/ml ± SD		
	Group 1, *n* = 50	Group 2, *n* = 40	Group 3 (control), *n* = 25
IFN-γ	0.98 ± 0.55*	0.87 ± 0.51*	0.44 ± 0.33
IL-4	0.29 ± 0.14	0.28 ± 0.14	0.24 ± 0.12
IL-10	1.82 ± 1.28	1.72 ± 1.28	1.58 ± 0.97
IL-17A	4.93 ± 1.54*	4.87 ± 1.57*	1.93 ± 0.83

*Significant difference in cytokines levels between the patients and healthy subjects

Levels of TNF-α, IL-1β and IL-12, the cytokines produced by monocytes/macrophages, detected in the sera of patients (groups 1 and 2) and in healthy individuals (group 3) are presented in Table 2. Levels of TNF-α and IL-12 in both groups of the patients fitted the normal range. On the other hand, the mean levels of IL-1β in groups 1 and 2 amounted to 0.54 ± 0.25 pg/ml and 0.50 ± 0.25 pg/ml, respectively. They manifested no significant differences between groups 1 and 2, but they were significantly higher than the values in the control group.

**Table 2:** Cytokines levels produced by monocytes/macrophages (TNF-α, IL-1β, IL-12) in the sera of patients with chronic suppurative dermatitis (group 1), with chronic inflammation in the upper respiratory tract (group 2) and in the control group (group 3)

Studied cytokine	Mean values in pg/ml ± SD		
	Group 1, *n* = 50	Group 2, *n* = 40	Group 3 (control), *n* = 25
TNF-α	0.77 ± 0.39	0.74 ± 0.3	0.61 ± 0.36
IL-1β	0.54 ± 0.25*	0.50 ± 0.25*	0.26 ± 0.14
IL-12	0.45 ± 0.35	0.42 ± 0.33	0.38 ± 0.31

*Significant difference in cytokines levels between the patients and healthy subjects

The diversification of individual Agr classes among the *S. aureus* strains isolated from the patients is presented in Table 3. In groups 1 and 2, the proportion of *S. aureus* strains with Agr IV phenotype amounted to 58 % and 65 %, respectively, and such a phenotype was significantly more frequent than the remaining phenotypes of Agr I–III. In parallel, no significant difference in the diversification of Agr classes could be demonstrated between groups 1 and 2 (*p* > 0.05).

**Table 3:** Diversification of Agr system classes among *S. aureus* isolates obtained from the examined patients of groups 1 and 2

Agr classes	Group 1, *n* = 50	Group 2, *n* = 40	*p*-value for difference between groups 1 and 2
I	12 (24 %)	8 (20 %)	*p* = 0.6513
II	5 (10 %)	3 (7.5 %)	*p* = 0.6798
III	4 (8 %)	3 (7.5 %)	*p* = 0.8588
IV	29 (58 %)*	26 (65 %)*	*p* = 0.5003

*Difference in the frequency of manifestation, as compared to Agr classes I, II and III, at the level of *p* < 0.001

In the analysis of cytokine response profiles in the context of the isolated strains of *S. aureus* in each of the patient groups (group 1 and group 2), respective subgroups of 1a and 2a were distinguished, linked to the isolated *S. aureus* strains manifesting the phenotype of Agr I–III, and subgroups 1b and 2b, linked to *S. aureus* strains manifesting Agr IV phenotype. Levels of IFN-γ, IL-4, IL-10 and IL-17A detected in the sera of patients in the subgroups 1a, 1b, 2a and 2b are presented in Table 4. The obtained levels of the studied cytokines manifested no significant differences between subgroups 1a and 1b or between subgroups 2a and 2b (*p* > 0.05). Levels of TNF-α, IL-1β and IL-12, detected in the sera of patients in subgroups 1a, 1b, 2a and 2b are presented in Table 5. Levels of the examined cytokines manifested no significant differences between subgroups 1a and 1b or between subgroups 2a and 2b (*p* > 0.05).

**Table 4:** Cytokine levels produced by lymphocytes Th (IFN-γ, IL-4, IL-10, IL-17A) in the sera of patients in subgroups 1a and 2a, linked to *S. aureus* isolates manifesting Agr I–III phenotypes, and subgroups 1b and 2b, linked to *S. aureus* strains manifesting Agr IV phenotype

Studied cytokine	Mean values in pg/ml ± SD			
	Group 1, *n* = 50		Group 2, *n* = 40	
	Subgroup 1a: Agr I–III, *n* = 21	Subgroup 1b: Agr IV, *n* = 29	Subgroup 2a: Agr I–III, *n* = 14	Subgroup 2b: Agr IV, *n* = 26
IFN-γ	1.01 ± 0.59	0.96 ± 0.52	0.81 ± 0.49	0.91 ± 0.52
IL-4	0.38 ± 0.16	0.32 ± 0.14	0.31 ± 0.14	0.33 ± 0.15
IL-10	1.84 ± 1.17	1.81 ± 1.37	1.79 ± 1.27	1.68 ± 1.31
IL-17A	5.1 ± 1.66	4.81 ± 1.47	4.98 ± 1.75	4.82 ± 1.5

**Table 5:** Cytokines levels produced by monocytes/macrophages (TNF-α, IL-1β, IL-12) in the sera of patients in subgroups 1a and 2a, linked to *S. aureus* isolates manifesting Agr I–III phenotypes, and subgroups 1b and 2b, linked to *S. aureus* strains manifesting Agr IV phenotype

Studied cytokine	Mean values in pg/ml ± SD			
	Group 1, *n* = 50		Group 2, *n* = 40	
	Subgroup 1a: Agr I–III, *n* = 21	Subgroup 1b: Agr IV, *n* = 29	Subgroup 2a: Agr I–III, *n* = 14	Subgroup 2b: Agr IV, *n* = 26
TNF-α	0.68 ± 0.36	0.84 ± 0.4	0.82 ± 0.36	0.7 ± 0.26
IL-1β	0.48 ± 0.26	0.58 ± 0.24	0.47 ± 0.26	0.52 ± 0.25
IL-12	0.42 ± 0.36	0.48 ± 0.35	0.37 ± 0.34	0.45 ± 0.33

## Discussion

This study analysed the levels of the circulating cytokines secreted by subpopulations of Th lymphocytes and monocytes/macrophages in patients with *S. aureus* chronic infections, and diversified classes of Agr systems among the isolated clinical strains of *S. aureus*. IFN-γ, mainly produced by activated Th1 lymphocytes, provides an optimal help for cellular immunity, including pronounced stimulation of monocytes/macrophages [13, 14]. On the other hand, IL-4 and IL-10, produced mainly by activated Th2 lymphocytes, provide optimal help for humoral immune response, while they suppress the reactions controlled by Th1 cells and, in parallel, they diminish pro-inflammatory functions of monocytes/macrophages [13, 15]. IL-17, in turn, produced mainly by activated Th17 lymphocytes, represents a pro-inflammatory cytokine, playing a significant role in the induction of local or systemic immune reactions, by firstly recruiting neutrophils against infectious agents [16, 17]. Classical pro-inflammatory cytokines, TNF-α, IL-1β and IL-12, produced mainly by activated monocytes/macrophages, demonstrate a broad immunostimulatory activity and, in parallel, they cooperate with IL-17 in the induction of inflammatory host reactions [18].

In this study, elevated levels of IFN-γ and IL-17A were documented, but IL-4 and IL-10 levels were within the normal ranges in the sera of patients with *S. aureus* infections, manifested by chronic suppurative dermatitis or chronic inflammation in the upper respiratory tract. The results point to a continuous response of Th1/Th17 lymphocytes, accompanied by a failed induction of Th2 cells in the course staphylococcal chronic infection. In part, the conclusion corroborates the earlier studies of Breuer et al. [19] and, recently, those of Niebuhr et al. [20], who showed that α-toxin, the 33-kDa cytolysin secreted by *S. aureus*, induced the release of IFN-γ and IL-17 in human CD4^+^ T cells. Moreover, staphylococcal peptidoglycan (PGN), with mediation of dendritic cells (DCs), was noted to induce Th1/Th17 response [21]. In addition, in the most common inflammatory human skin diseases, atopic dermatitis [22] and psoriasis [23], an increase was noted in circulating Th1 and Th17 cells. Apart from the distinct lines of Th1 and Th17 lymphocytes, also the mixed Th17/Th1 cell type is distinguished, which combines the pro-inflammatory potential of Th17 and Th1 cells, being characterised by the production of both IFN-γ and IL-17 [24]. These Th17/Th1 cells manifest a stable coexpression of transcription factors T-bet and RORγt, most probably originating from Th17 lymphocytes, and account for a rare population of circulating cells, representing only 0.05–0.11 % of CD4^+^ T cells in human healthy blood [25, 26]. In the context of the data, the increased levels of IFN-γ and IL-17A detected by us in the sera of patients may reflect a preferential induction and accumulation of Th17/Th1 cells in the foci of dermatitis or upper respiratory mucositis induced by *S. aureus* infection. Secretory activity of the cells is probably involved in the development of chronic inflammatory processes, linked to *S. aureus* infection. This potential has been demonstrated by in vitro studies of Boniface et al. [27], showing that, in contrast to human Th1 or Th17 lymphocytes, Th17/Th1 cells manifest a greater propensity to induce inflammation on epithelial cells. On the other hand, Cho et al. [28] demonstrated, using a mouse model, that IL-17, priming neutrophil recruitment, is ultimately required for host defence against *S. aureus* skin infections. The 2.5-fold increase in circulating IL-17A detected by us in patients is apparently insufficient for the induction of an adequate response against *S. aureus* infection but, together with the released IFN-γ, it may promote the development of a chronic inflammatory process. In our study, we have demonstrated a doubled increase in levels of circulating IFN-γ, which, however, was not accompanied by increased levels of circulating TNF-α and IL-12. In turn, the augmented level of circulating IL-1β detected by us might have resulted from secretion of the cytokine by IFN-γ stimulated cells of vascular endothelium [29]. Thus, the demonstrated increased levels of IFN-γ are insufficient for an effective induction of secretory function in monocytes/macrophages. In parallel, studies of Minegishi et al. [30] documented an immunological role of anti-staphylococcal factors, including neutrophil-recruiting chemokines and antimicrobial peptides, the secretion of which by human keratinocytes and bronchial epithelial cells is deeply dependent on the synergistic action of IL-17 and the combination of other pro-inflammatory cytokines (TNF-α + IL-1β + IFN-γ). In that context, it seems probable that the development of infection with *S. aureus* in the studied patients was linked to an insufficient stimulation of epithelial cell secretory function in the skin and upper respiratory tract due to impoverished cytokine response of monocytes/macrophages. In parallel, the preferentially pathogen-induced lymphocytes Th17/Th1 may be responsible for the promotion of chronic dermatitis or chronic respiratory tract infection. Quite possibly, *S. aureus* strains with deficient production of delta-toxin are responsible for the preferential induction, as it has seemed to be indicated by recent studies of Gagnaire et al. [31]. In turn, Zielinski et al. [32] have found that, due to a continuous stimulation by a pathogen, Th17 cells may acquire the ability to produce IL-10. In parallel, the above-quoted authors have shown that the phenomenon is inhibited by IL-1β. The data correspond to the increased levels of IL-1β demonstrated by us in this study.

Among the isolated strains of *S. aureus*, originating either from patients with suppurative infections or from patients with upper respiratory tract inflammation, we have demonstrated a significant dominance of class IV Agr, while diversification of the remaining Agr classes demonstrated no significant differences. The dominance of class IV Agr among strains isolated from Polish patients, mainly patients with suppurative infections, has been described previously [33]. Moreover, strains with class IV Agr were found to be rare among healthy carriers of *S. aureus* [33, 34]. A relationship was also described between strains of *S. aureus* belonging to class IV Agr and the production of epidermolytic toxins (exfoliatins, ETs) and the phylogenetic group AF1, in contrast to AF2, which corresponded to strains of class I and II Agr, and AF3, which corresponded to strains of class III Agr [10, 35]. In this study, however, in none of the patients could generalised or localised forms of staphylococcal scalded skin syndrome (SSSS) be diagnosed, induced by ETs [36]. Thus, the clinical data indicate that the strains isolated from *S. aureus* patients produced no ETs. Moreover, the sera of studied patients demonstrated no extremely high levels of IFN-γ, IL-17A or IL-1β and concentrations of TNF-α remained within the normal range. It is already well recognised that the toxins released by *S. aureus*, including enterotoxins, toxic shock syndrome toxin-1 and ETs, exert activity of superantigens on the host immune system [36, 37]. Such an activity results in extremely high levels of circulating various cytokines, including TNF-α [37, 38].

Upon analysis of the studied profile of cytokine response, we have demonstrated no qualitative or quantitative differences between subgroups of patients linked to infections with *S. aureus* strains belonging to class IV Agr and patients infected with strains belonging to the remaining classes I–III Agr. The data allow to suggest that the antigenic profile of virulence factors released by *S. aureus* does not depend on the Agr class of the pathogen. The suggestion corroborates the earlier studies of Jarraud et al. [10], pointing to an absence of a direct role of the Agr class in the type of human disease induced by *S. aureus*. Nevertheless, in contrast to healthy carriers of *S. aureus*, strains isolated from patients are dominated by those belonging to class IV Agr. Thus, it seems possible that this Agr phenotype is particularly linked to the activation of virulence factors, which preconditions its preferential relationship with the pathogenicity of *S. aureus*.

Thus, our results demonstrate that patients with *S. aureus* infections, manifested by chronic suppurative dermatitis or chronic inflammation of the upper respiratory tract, develop a Th17/Th1 cytokine response, not accompanied by a sufficient pro-inflammatory response of monocytes/macrophages. Therefore, it is possible that lymphocytes Th17/Th1 are responsible for the promotion of a chronic inflammatory process induced by *S. aureus*. In parallel, it was demonstrated that cytokine response does not differ between subgroups of patients infected with *S. aureus* strains belonging to class IV Agr and those infected with strains belonging to the remaining classes I–III Agr. In patients, the strains of class IV Agr prevailed, which points to a preferential relationship between the class and pathogenicity of *S. aureus*.
